# Morphology Control of Spinel LiNi_0.5_Mn_1.5_O_4_ for Tuned Microstructure and Electrochemistry

**DOI:** 10.3390/ma19163507

**Published:** 2026-08-19

**Authors:** Jingjun Liu, Mingliang Yuan, Hailong Liu, Zetong Fan, Ming Zhang, Sitong Du

**Affiliations:** 1 Department of Materials Engineering, Taiyuan Institute of Technology, Taiyuan 030008, China13546077820@163.com (M.Z.); 15835913318@163.com (S.D.); 2 School of Mineral Processing and Bioengineering, Central South University, Changsha 410083, China

**Keywords:** lithium-ion batteries, high-voltage spinel cathode, primary particles, electrochemical performance

## Abstract

**Highlights:**

- Morphology-tuned octahedral LNMO shows remarkable cycling stability at 25 °C and 55 °C.
- Submicron primary particles curb side reactions and greatly accelerate lithium ion diffusion.
- Sol–gel octahedral particles feature high crystallinity, few defects, and low inter-facial impedance.
- Morphology engineering alleviates intrinsic flaws of cobalt-free high-voltage spi-nel cathode materials.
- This tunable sol–gel route enables scalable synthesis of high-performance octahe-dral LNMO cathodes.
- Optimized octahedral LNMO fits long-life high-power batteries for electric vehi-cles and energy storage.

**Abstract:**

High-voltage spinel LiNi_0.5_Mn_1.5_O_4_ (LNMO) is a promising cathode for next-generation lithium-ion batteries, yet its application is limited by structural instability and poor high-temperature/high-rate performance. Here, spherical secondary polycrystalline aggregate (LNMO-PC), micron-sized primary particle (single-crystal morphology) (LNMO-SC-L), and submicron primary particle (single-crystal morphology) (LNMO-SC-S) LNMO were synthesized. Their structures, morphologies, and surface properties were characterized and electrochemical performance evaluated at room (25 °C)/high (55 °C) temperature and high rates. LNMO-SC-S exhibited the highest crystallinity, lowest Mn^3+^ content, and minimal charge-transfer resistance. It showed superior cycling stability (93.3% retention at 1 C over 500 cycles), excellent rate capability (~20 mAh g^−1^ at 20 C), and enhanced high-temperature performance. The submicron primary particles suppress grain-boundary degradation and Mn^3+^ disproportionation, shortens Li^+^ paths, and improves reaction kinetics.

## 1. Introduction

The demand for high-energy-density lithium-ion batteries (LIBs) in electric vehicles, grid-scale energy storage systems, and portable electronics continue to surge, driving intensive efforts to develop advanced cathode materials that simultaneously deliver high-rate capability, ultralong cycle life, and robust thermal stability. Among the candidates, high-voltage spinel lithium nickel manganese oxide (LNMO) has emerged as one of the most promising cathodes for next-generation LIBs, owing to its exceptionally high operating voltage (~4.7 V vs. Li/Li^+^), considerable theoretical specific capacity (~147 mAh g^−1^), favorable kinetic properties, and cost advantages. Nevertheless, the large-scale commercialization of LNMO remains strongly constrained by several critical bottlenecks, including rapid capacity fading during prolonged cycling, inadequate high-temperature stability, and pronounced electrochemical polarization. These challenges largely originate from the intrinsic structural instability of LNMO and frequent parasitic reactions at the electrode–electrolyte interface.

The synthesis of LiNi_0.5_Mn_1.5_O_4_ involves a wide range of processes, mainly divided into two categories: solid-phase methods and liquid-phase methods. Solid-phase methods include molten salt synthesis, high-temperature solid-state reactions, and mechanical activation; liquid-phase methods encompass hydrothermal synthesis, co-precipitation, sol–gel synthesis, composite carbonate methods, and spray pyrolysis. Different preparation methods can result in LNMO materials with significant differences in particle size, crystal structure, and microstructure [1]. Solid-phase methods are the most widely used and involve grinding or ball milling stoichiometric raw materials followed by high-temperature heat treatment. In contrast, liquid-phase wet chemical synthesis routes, such as sol–gel and co-precipitation, offer advantages in precisely controlling particle size, morphology, and dispersion uniformity. Synthesis parameters are critical in determining both the production cost and the final performance of the materials. Both research and industrial sectors focus on factors such as raw material type, synthesis method [2,3], calcination temperature [4], calcination time [5], and calcination atmosphere [6]. The preparation process directly determines the microstructure of the material, which in turn governs its electrochemical performance.

To further optimize the performance of LNMO, researchers have developed various preparation methods that can directionally control the microstructure. Takahiro Kozawa et al. [7] employed a low-temperature steam-assisted method to synthesize LNMO, where steam promotes grain growth, yielding cathode materials with more regular morphology and superior performance compared to those synthesized in air atmosphere. Xue et al. [8] controlled the particle size and dispersion of LNMO by adjusting the rate of precipitant addition, with rapid addition reducing particle size, achieving optimal electrochemical performance when the particle size was controlled at 8 μm. Chang et al. [9] utilized a two-stage roasting process to produce LNMO with high crystallinity and structural stability, achieving an initial capacity of 130.3 mAh/g, with a capacity retention of 96.7% after 100 cycles (1.0 C). Han et al. [10] synthesized structurally stable LNMO by modifying lattice sites, enhancing intrinsic particle kinetics, reducing charge transfer barriers, and increasing Li^+^ transport rates. Zeng et al. [11] used polyethylene oxide (PEO) as a soft template to construct hollow LNMO microspheres with a three-dimensional channel structure, where PEO facilitated uniform nucleation of the precursor. This unique hollow morphology significantly improved the cycling and rate performance. Miao et al. [12] synthesized spherical hollow LNMO via a glucose-assisted hydrothermal method, where the hollow spherical morphology drastically shortened the Li^+^ transport path and enhanced lithium-ion storage performance. Liang et al. [13] employed an ethanol gel method combined with segmented sintering to produce micron-sized single-crystal LNMO, with the single-crystal morphology showing no grain boundary defects, resulting in significantly improved capacity retention and thermal stability. Nisar et al. [14] used microwave-assisted synthesis to obtain LNMO with uniform crystal structure, significantly reducing the charge transfer resistance at the electrode/electrolyte interface. Haridas et al. [15] applied a polymer-assisted sol–gel/electrospinning method to produce worm-like spinel LNMO with an Fd-3m space group, where this unique one-dimensional morphology provided excellent high-rate performance and cycling stability. Ma et al. [16] utilized various MnO_2_ precursors and a template method to prepare LNMO with diverse morphologies, confirming that the precursor directly determines the material’s microstructure and consequently its electrochemical performance. Liu et al. [4] synthesized LNMO microspheres with octahedral nanoparticle structures using a co-precipitation method, with the multi-level microspherical morphology imparting excellent electrochemical properties to the material. Lee et al. [17] synthesized LNMO using a sol–gel method at 600–1000 °C and found that calcination temperature could regulate the crystallinity and phase structure, with the product predominantly featuring an Fd-3m disordered phase and a minor amount of the ordered P4_3_32 phase, further demonstrating the structure–performance relationship between temperature, morphology, and properties.

Based on the aforementioned research background, this study aims to systematically investigate the mechanisms by which morphology control affects the crystal structure, microstructure, and electrochemical performance of LNMO. Three types of samples—secondary polycrystalline aggregate LNMO (LNMO-PC), micron-sized primary particle (single-crystal morphology) LNMO (LNMO-SC-L), and submicron primary particle (single-crystal morphology) LNMO (LNMO-SC-S)—are prepared using co-precipitation, molten salt, and sol–gel methods, respectively. The crystal ordering, microstructure, and surface chemical states of the samples are analyzed using characterization techniques such as X-ray diffraction (XRD), scanning electron microscopy (SEM), Fourier transform infrared spectroscopy (FTIR), Raman spectroscopy, X-ray photoelectron spectroscopy (XPS), and particle size distribution curves. Electrochemical performance is systematically evaluated at room temperature (25 °C), elevated temperature (55 °C), and high rates (up to 20 C) through constant current charge/discharge, cyclic voltammetry (CV), and electrochemical impedance spectroscopy (EIS) tests. It should be noted that the terms “single-crystal morphology” and “polycrystalline morphology” adopted in this paper follow the conventional naming habit of lithium-ion battery cathode research rather than the strict crystallographic definition judged by single diffraction spots under TEM observation. In the field of spinel LiNi_0.5_Mn_1.5_O_4_ materials, octahedral primary particles with smooth surfaces and negligible intergranular cracks are generally defined as “single-crystal morphology particles”, while spherical secondary aggregates stacked by abundant tiny primary crystallites are named “polycrystalline morphology particles”. There is inherent coupling between the external particle morphology and internal crystallographic parameters, including crystallite size, cation disorder, and Mn valence distribution, which is the core research object of this work.

The results show that the morphological characteristics of LNMO play a decisive role in its structural stability and electrochemical performance. Submicron primary particle (single-crystal morphology) LNMO (LNMO-SC-S) exhibits the highest degree of structural order, lowest Mn^3+^ content, and the smallest charge transfer resistance (57.29 Ω) while demonstrating the best cycling stability (capacity retention of 93.28% after 500 cycles at 1 C rate at 25 °C) and rate performance (approximately 20 mAh g^−1^ at 20 C rate). Its superior performance is attributed to the effect of the submicron primary particle (single-crystal morphology) structure: it suppresses the Mn^3+^ disproportionation reaction and grain boundary degradation, while shortening the lithium-ion diffusion path and enhancing electron transport rate. In contrast, spherical polycrystalline aggregate morphology LNMO (LNMO-PC), due to its dense grain boundary defects and higher Mn^3+^ content, shows the most severe capacity fade and polarization. Micron-sized primary particle LNMO (LNMO-SC-L) has intermediate performance, with its rate capability limited by the longer solid-phase lithium-ion diffusion path. Table 1 compares the cycling stability, charge transfer resistance (Rct), and high-temperature performance of as-prepared LNMO-SC-S with various coated LNMO cathodes from previous reports. Most reported high-performance LNMO materials rely on extra surface coating or composite modification, yet they are only tested under mild low-rate and short-cycle conditions, lacking systematic high-rate and long-term high-temperature cycling data. The LiCoPO_4_, graphene, and LiPON-modified samples exhibit much larger charge transfer resistance than LNMO-SC-S. After 200 cycles, our LNMO-SC-S only delivers an Rct of 57.29 Ω, which guarantees fast Li^+^ migration and low polarization. Without any coating treatment, LNMO-SC-S achieves 93.28% capacity retention after 500 cycles at 1 C and negligible decay over 200 cycles at 5 C, showing excellent durability at both low and high rates. Moreover, it maintains 74.92% capacity retention after 200 cycles at 55 °C, a harsh high-temperature test condition that most reference samples have not covered. This comparison confirms that morphology regulation to fabricate submicron primary particles is a simple and efficient strategy to comprehensively boost the electrochemical properties of LNMO, eliminating complicated coating processes used in other studies.

For clarity of data sources, all characterizations of LNMO-SC-L and LNMO-PC were newly measured in this work. The SEM, XPS, and CV data of benchmark LNMO-SC-S are reused from our prior papers [22,23] with Elsevier reprint permissions detailed in the corresponding figure captions. Unlike our earlier doping and annealing studies, this work focuses on morphology-dependent structure–performance correlations; LNMO-SC-S merely serves as a reference, and the comparison framework and mechanistic analysis here are independently developed.

## 2. Materials and Methods

All chemical reagents were used as received without further purification.

Lithium acetate dihydrate Li(CH_3_COO)⋅2H_2_O (analytical grade, Macklin Biochemical Co., Ltd., Shanghai, China); nickel acetate tetrahydrate Ni(CH_3_COO)_2_⋅4H_2_O (analytical grade, Macklin Biochemical Co., Ltd., Shanghai, China); manganese acetate tetrahydrate Mn(CH_3_COO)_2_⋅4H_2_O (analytical grade, Titan Scientific Co., Ltd., Shanghai, China); citric acid monohydrate C_6_H_8_O_7_⋅H_2_O (analytical grade, Aladdin Biochemical Technology Co., Ltd., Shanghai, China); lithium chloride LiCl (analytical grade, Aladdin Biochemical Technology Co., Ltd., Shanghai, China); potassium chloride KCl (PT grade, Macklin Biochemical Co., Ltd., Shanghai, China); manganese sulfate heptahydrate MnSO_4_⋅7H_2_O (analytical grade, Sinopharm Chemical Reagent Co., Ltd., Beijing, China); nickel sulfate hexahydrate NiSO_4_⋅6H_2_O (99% purity, Macklin Biochemical Co., Ltd., Shanghai, China); anhydrous sodium carbonate (analytical grade, Sinopharm Chemical Reagent Co., Ltd., Beijing, China); ammonia water NH_3_⋅H_2_O (25 vol%, Zhiyuan Chemical Reagent Co., Ltd., Tianjin, China); and deionized water were adopted for material synthesis.

### 2.1. Synthesis of Materials

The process for preparing submicron primary particle (single-crystal morphology) LiNi_0.5_Mn_1.5_O_4_ was as follows: Metal salts with a molar ratio Li:Ni:Mn = 2.1:1:3 were dissolved in 105 mL deionized water to form a homogeneous metal precursor solution. Citric acid was added as chelating agent at a molar ratio of total metal ions: citric acid = 1:1, dissolved in 40 mL deionized water. The citric acid solution was dropwise added into metal salt solution under continuous stirring, and ammonia water was used to adjust the mixed solution pH to 7. The mixture was stirred at 50 °C with a stirring speed of 500 rpm for 4 h in a thermostatic water bath (DK-98-IIA, Taiste Instrument Co., Ltd., Tianjin, China, Same equipment hereafter). Afterwards, the temperature was raised to 80 °C and maintained for 2.5 h at 500 rpm, then stirred at 360 rpm for another 2.5 h to evaporate most solvent and form wet gel. The wet gel was dried overnight at 120 °C in a blast drying oven (DHG-9076A, Jinghong Instrument Co., Ltd., Shanghai, China, same equipment hereafter) and manually ground to obtain dry precursor powder. The precursor was calcined in a muffle furnace (KSL-1200X, Kejing Materials Technology Co., Ltd., Shenzhen, China, same equipment hereafter) at a heating rate of 3 °C min^−1^ up to 450 °C and held for 5 h, followed by natural cooling and re-grinding. The powder was then heated to 850 °C at 3 °C min^−1^ and sintered for 12 h. After furnace cooling and grinding, submicron primary particle LNMO powder was obtained [22].

The process for preparing micron-sized primary particle (single-crystal morphology) LiNi_0.5_Mn_1.5_O_4_ was as follows: First, a certain amount of nickel acetate and manganese acetate, in a stoichiometric ratio of 1:3, was dissolved in deionized water and stirred until completely dissolved. Then, LiCl and KCl low-melting-point salts were weighed in a 59:41 ratio, ensuring that the ratio of the solvent to the metals in the raw materials was 1:50. After mixing, the mixture was stirred, evaporated, and ground to obtain the precursor. The precursor was placed in a muffle furnace, heated at a rate of 3 °C/min to 450 °C, and pre-sintered for 5 h. After cooling, it was ground and then heated at 3 °C/min to 850 °C for 8 h. After natural cooling and grinding, the final large single-crystal morphology LNMO product was obtained.

The process for preparing secondary polycrystalline aggregate LiNi_0.5_Mn_1.5_O_4_ was as follows: Manganese sulfate monohydrate and nickel sulfate hexahydrate were dissolved in deionized water in a Mn/Ni molar ratio of 3:1 to prepare a mixed metal salt solution. Anhydrous sodium carbonate solution was used as a precipitant, which was slowly and uniformly added to the metal salt solution using a constant flow titration pump (BT600-2J, Longer Pump Co., Ltd., Baoding, China). The solution was stirred continuously at a constant temperature to ensure the precipitation reaction proceeds fully. After completion of the reaction, the mixture was vacuum filtered, washed to remove impurities, and dried at 80 °C to obtain a nickel–manganese composite carbonate precursor powder Ni_0.25_Mn_0.75_CO_3_ with a fixed transition metal stoichiometry of Ni:Mn = 1:3. The precursor Ni_0.25_Mn_0.75_CO_3_ was then mixed with lithium salt in a 1:1.1 stoichiometric ratio (with excess lithium salt to compensate for lithium loss during high-temperature calcination). The mixture was subjected to a segmented heat treatment in an air atmosphere, first pre-sintered at 500 °C for 5 h, then sintered at 850 °C for 8 h. After annealing, cooling, and thorough grinding, black secondary polycrystalline aggregate LNMO powder was obtained.

### 2.2. Morphological and Structural Characterization of Materials

A comprehensive structural and morphological characterization of the samples was conducted. X-ray powder diffraction (XRD, TD3500, Tongda Instrument Co., Ltd., Dandong, China), Fourier transform infrared spectroscopy (FTIR, Nicolet iS5, Thermo Fisher Scientific, Waltham, MA, USA), and Raman spectroscopy (inVia, Renishaw, Gloucestershire, UK) were used to analyze the crystal structure and chemical bonding characteristics of the materials. Scanning electron microscopy (SEM, MIRA4 LMH, TESCAN, Brno, Czech Republic) was employed to observe the microstructure and exposed crystal facets of the samples. X-ray photoelectron spectroscopy (XPS, K-Alpha+, Thermo Fisher Scientific, Waltham, MA, USA) was used to analyze the chemical states of surface elements. Additionally, Laser particle size analysis was carried out on a Mastersizer 2000 instrument (Malvern Panalytical, Malvern, UK) to record volume-weighted particle size distribution curves of each sample. Inductively coupled plasma optical emission spectrometry (ICP- OES, 5110, Agilent Technologies, Santa Clara, CA, USA) was utilized to measure actual Li/Ni/Mn stoichiometric ratios of three samples, quantifying lithium volatilization loss, and elemental deviation after high-temperature calcination.

### 2.3. Assembling and Electrochemical Testing of Coin Cells

Electrode slurries were prepared by mixing LNMO active material, conductive carbon black (battery grade, Shuotian Technology Co., Ltd., Shenzhen, China) and polyvinylidene fluoride (PVDF, battery grade, Arkema, Paris, France) at a mass ratio of 8:1:1, with N-methylpyrrolidone (NMP, battery grade, Sinopharm Chemical Reagent Co., Ltd., Beijing, China) as solvent. The homogeneous slurry was coated on aluminum foil current collector (battery grade, Kejing Materials Co., Ltd., Hefei, China), dried, and calendared to obtain cathode sheets with consistent active mass loading. Identical calendering pressure and final electrode thickness were applied for all electrode slices. All electrode discs were punched to a uniform diameter of 14 mm. The measured active material loadings on Al foils are listed as follows: LNMO-SC-S (2.043 mg), LNMO-SC-L (2.018 mg), and LNMO-PC (2.103 mg). Standard CR2025 coin cells were assembled in an argon-filled glove box (ZK-82B, Mikrouna, Shanghai, China, O_2_ and H_2_O < 0.1 ppm). Lithium metal foil (battery grade, Beinuo Battery Materials Co., Ltd., Xinghua, China) served as counter electrode; polypropylene separator (battery grade, Lizhiyuan Battery Sales Department, Taiyuan, China) and commercial electrolyte (1 M LiPF_6_ dissolved in EC:DMC:EMC, battery grade, Saibo Electrochemical Reagent Co., Ltd., Tianjin, China) were used. Galvanostatic charge–discharge, rate, and high-temperature cycling tests were conducted on a battery test system (BTS-51, Neware Electronic Co., Ltd., Shenzhen, China) with voltage window 3.5–4.9 V (vs. Li/Li^+^). Cyclic voltammetry (CV) and electrochemical impedance spectroscopy (EIS) were tested on an electrochemical workstation (Interface 1000T, Gamry Instruments, Warminster, PA, USA): CV scan rate = 0.1 mV s^−1^; EIS frequency range = 0.01 Hz–100 kHz, AC perturbation amplitude = 5 mV. For reproducibility verification, three parallel coin cells were assembled and tested for each sample under identical conditions.

## 3. Results and Discussion

### 3.1. Morphology and Structural Analysis

**Figure 1:**
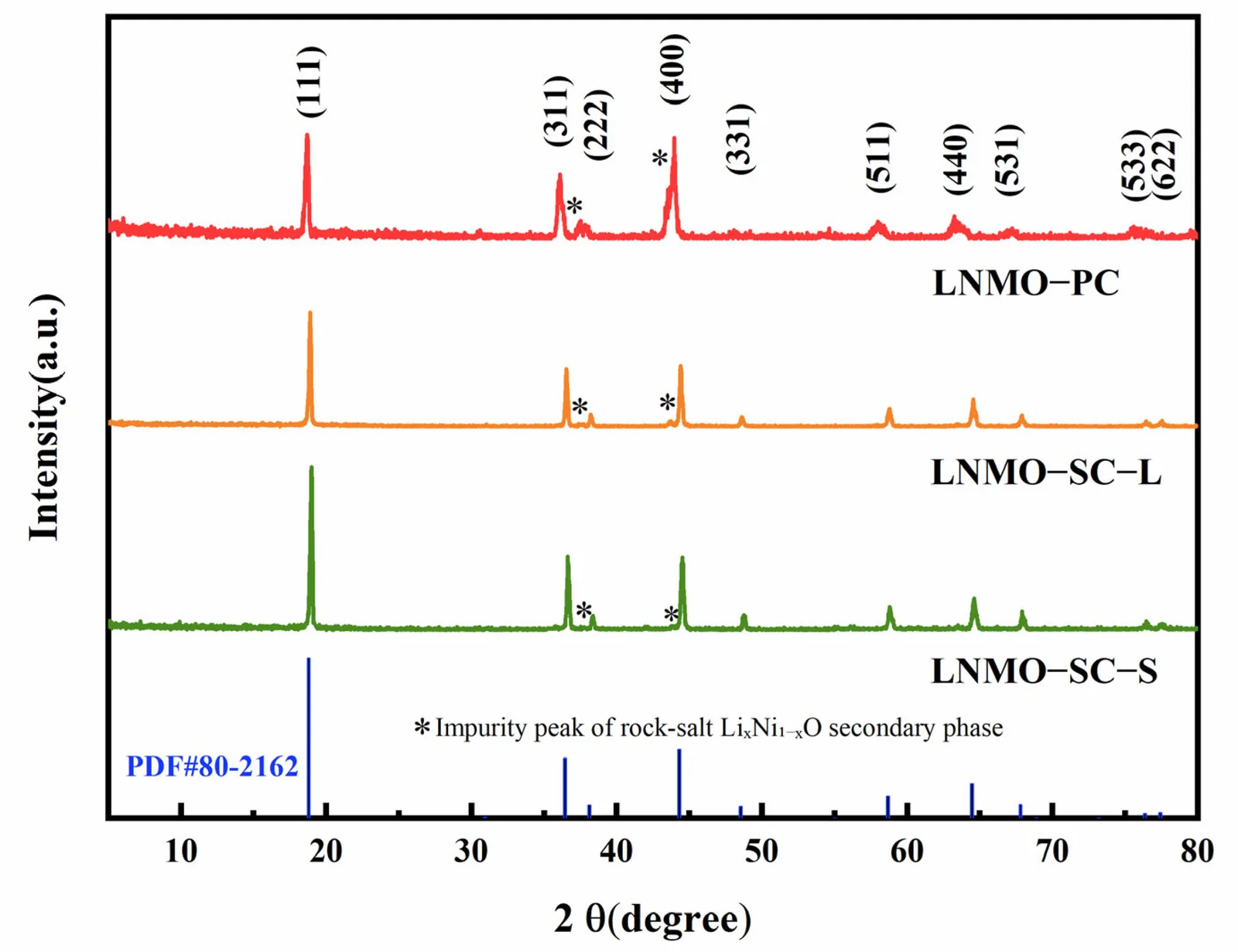
XRD patterns of LNMO-SC-S, LNMO-SC-L, and LNMO-PC.

The crystal structures of LNMO-PC, LNMO-SC-L, and LNMO-SC-S were systematically characterized by XRD (Figure 1). The diffraction peaks of all samples matched well with the spinel-type nickel manganese oxide standard card PDF#80-2162 (space group Fd-3m) [24], confirming the successful synthesis of the spinel main phase. Weak diffraction peaks marked with * correspond to rock-salt Li_x_Ni_1−x_O secondary impurity, whose peak intensity obeys LNMO-PC > LNMO-SC-L > LNMO-SC-S. Peak shifts and FWHM analysis further reveal structural discrepancies. Secondary polycrystalline aggregate LNMO-PC exhibits low-angle peak shifts and the broadest diffraction peaks, indicative of severe lattice expansion and grain-boundary-induced internal stress. LNMO-SC-L shows milder peak shifts and narrower peaks with reduced lattice strain. LNMO-SC-S matches the standard card almost perfectly with negligible peak offset, demonstrating minimal lattice distortion and superior atomic ordering. This structural feature effectively restrains lattice deformation and phase transition during cycling, contributing to outstanding electrochemical stability.

Full-spectrum Rietveld refinement was performed for three LNMO samples, with structural parameters summarized in Table 2. Low R_wp_ values (8.1–8.5%) verify reliable fitting results. Refined lattice constant a and unit cell volume decrease in the order LNMO-PC > LNMO-SC-L> LNMO-SC-S. Abundant grain boundaries in LNMO-PC trigger severe tetrahedral Li/Ni mixing and abundant oxygen vacancies. Subsequent XPS confirms LNMO-PC possesses the highest Mn^3+^ content (80.32%); the larger ionic radius of Mn^3+^ causes lattice expansion, consistent with its low-angle peak shift observed by XRD. LNMO-SC-L displays intermediate lattice dimensions matching its moderate Mn^3+^ proportion (75.67%). Low grain-boundary primary particle LNMO-SC-S has the lowest Mn^3+^ fraction (71.93%) and the smallest lattice volume. The I_311_/I_400_ ratio, characterizing harmful tetrahedral Li/Ni mixing, declines as LNMO-SC-S (1.0087) > LNMO-SC-L (0.9802) > LNMO-PC (0.6811). More grain boundaries accelerate cation interdiffusion and lower this ratio. Submicron primary particles efficiently restrain Li/Ni cation mixing and deliver superior lithium site ordering. Grain boundaries act as nucleation sites for rock-salt Li_x_Ni_1−x_O impurities, so impurity content ranks LNMO-PC > LNMO-SC-L > LNMO-SC-S. Excessive secondary phases in LNMO-PC induce irreversible capacity loss, while the continuous lattice of LNMO-SC-S inhibits impurity formation. In summary, particle morphology and grain boundary density govern structural differences across samples. Submicron primary particle (single-crystal morphology) LNMO-SC-S delivers highly ordered Li sites, minimal rock-salt impurities, and moderate Mn^3+^ originating from mild octahedral Ni/Mn disorder. Appropriate Mn^3+^ widens Li^+^ migration channels to boost rate performance without severe Mn disproportionation, which explains its superior charge transfer kinetics and long-cycle stability in subsequent electrochemical tests.

**Figure 2:**
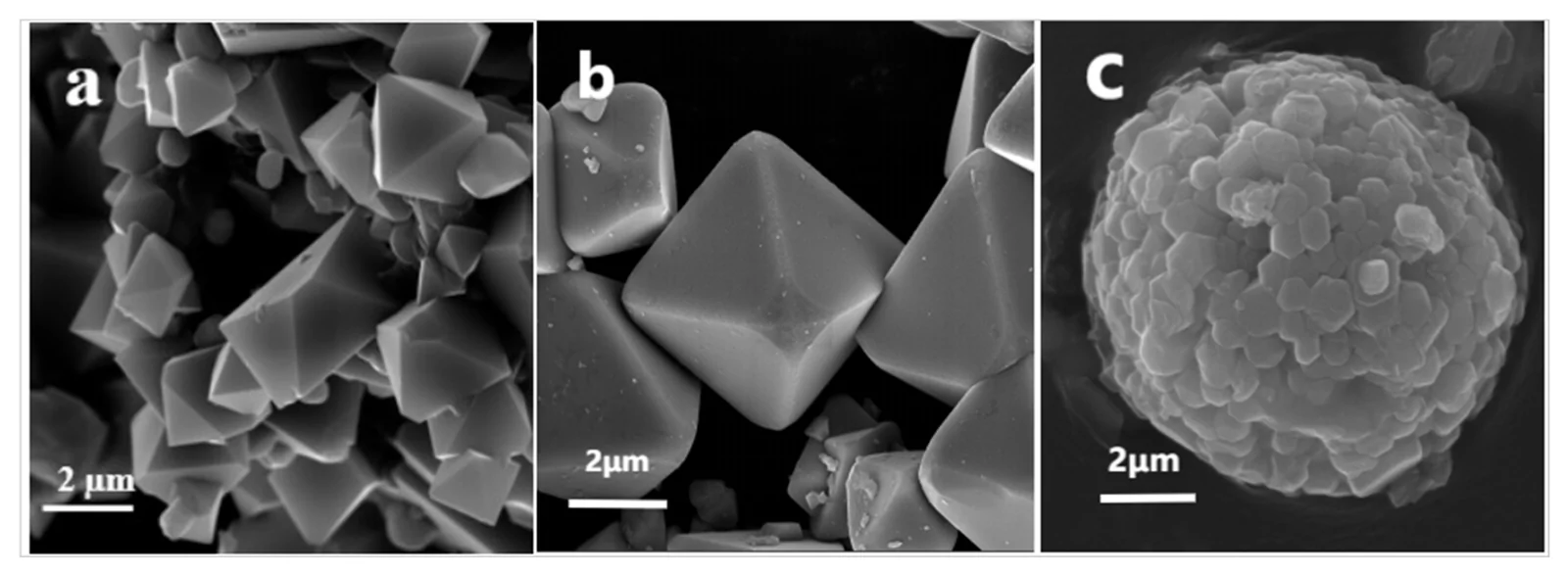
SEM images of (**a**) LNMO-SC-S [23] (reprinted from Journal of Alloys and Compounds, Vol. 971, Liu J, et al., Influence of different raw materials on the preparation of single-crystal LiNi_0.5_Mn_1.5_O_4_, Page 172778, Copyright (2024), with permission from Elsevier), (**b**) LNMO-SC-L, and (**c**) LNMO-PC.

As displayed in Figure 2a–c, three distinct particle morphologies are obtained from three synthetic routes, and obvious differences in particle exterior shapes can be observed. The sol–gel-synthesized LNMO-SC-S sample is composed of discrete, regular submicron primary particles with sharp crystal edges, smooth facets, and slight agglomeration, which is categorized as submicron primary particles in battery material studies. Such intact octahedral granular structure effectively shortens solid-state lithium-ion diffusion paths and mitigates concentration polarization; more importantly, this low grain-boundary primary particle avoids brittle fracture during electrode rolling and cycling volume expansion, maintaining stable ion/electron conductive networks as reported in ref. [25]. The molten-salt product LNMO-SC-L also exhibits well-developed octahedral single-crystal morphology with much larger particle size and denser outer surfaces. The liquid molten salt medium promotes sufficient surface ion migration and complete crystal plane reconstruction during sintering, eliminating tiny surface pits, gaps, and micro-protrusions, thus forming a compact, defect-sparse outer surface. The molten salt medium facilitates complete crystal plane rearrangement and oriented crystal growth, yet the enlarged particle dimension prolongs lithium solid diffusion distance and restricts reaction kinetics compared with submicron octahedral particles. In contrast, LNMO-PC prepared via co-precipitation presents spherical polycrystalline aggregate morphology. These spherical secondary particles are assembled from abundant tiny primary crystallites, leading to rough surfaces and dense internal grain boundaries. A high density of grain boundaries brings extra interfacial resistance and tortuous Li^+^ migration channels. Meanwhile, the stacked granular structure tends to crack and pulverize under compression and cyclic volume variation, accelerating performance degradation, which is consistent with the mechanism described in ref. [26]. Different particle shapes intrinsically alter internal microstructural features, including grain boundary density, crystallite dimension, and lattice strain, and further produce divergent electrochemical behaviors. The subsequent XRD, FTIR, Raman, and XPS characterizations further quantify the structure–morphology correlation among the three morphologically distinct samples.

**Figure 3:**
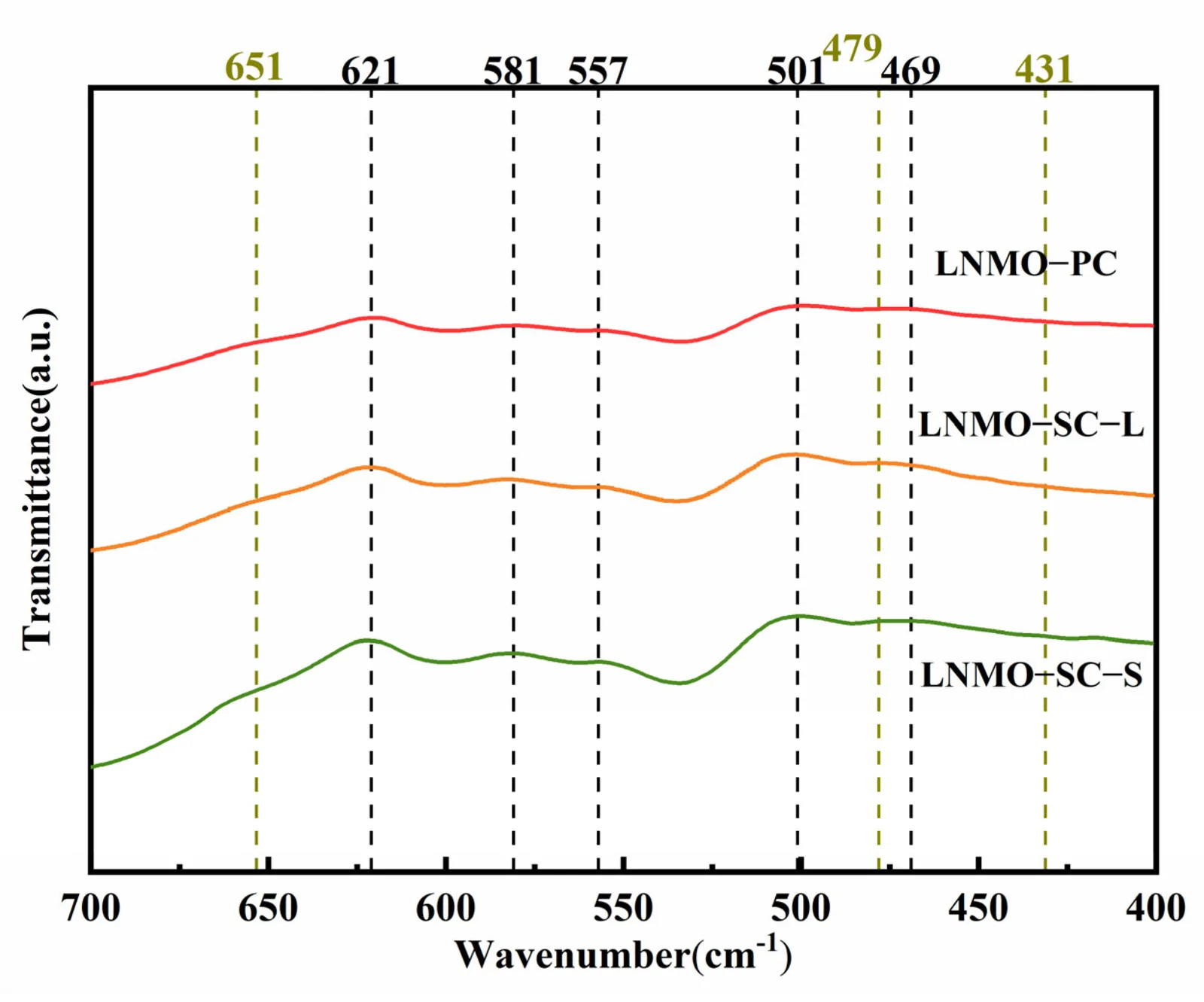
FTIR spectra of LNMO-SC-S, LNMO-SC-L, and LNMO-PC.

Based on FT-IR analysis (Figure 3), the three samples, LNMO-PC, LNMO-SC-L, and LNMO-SC-S, all exhibit characteristics of the Fd-3m space group, with absorption peaks at 621 cm^−1^, 581 cm^−1^, 557 cm^−1^, 501 cm^−1^, and 469 cm^−1^. The absorption peaks at 621 cm^−1^ and 557 cm^−1^ are associated with Mn–O vibrations, while the peaks at 581 cm^−1^ and 501 cm^−1^ correspond to Ni–O vibrations [16]. The absorption peak intensity of F-type LNMO materials is lower than that of P-type LNMO materials. The shapes and intensities of the absorption peaks of the three samples differ, reflecting changes in their structural order. In LNMO-PC, the absorption peaks at 621 cm^−1^ and 557 cm^−1^ broaden, indicating that this sample contains more lattice defects and impurity phases, resulting in higher disorder and lower crystallinity. LNMO-SC-L has sharper and more concentrated absorption peaks, indicating higher crystallinity and reduced disorder. LNMO-SC-S has the sharpest absorption peaks with no noticeable shoulder peaks and also exhibits characteristic peaks of the P4_3_32 space group at 651, 479, and 431 cm^−1^, indicating the highest crystallinity, lowest disorder, and most ordered structure [4].

**Figure 4:**
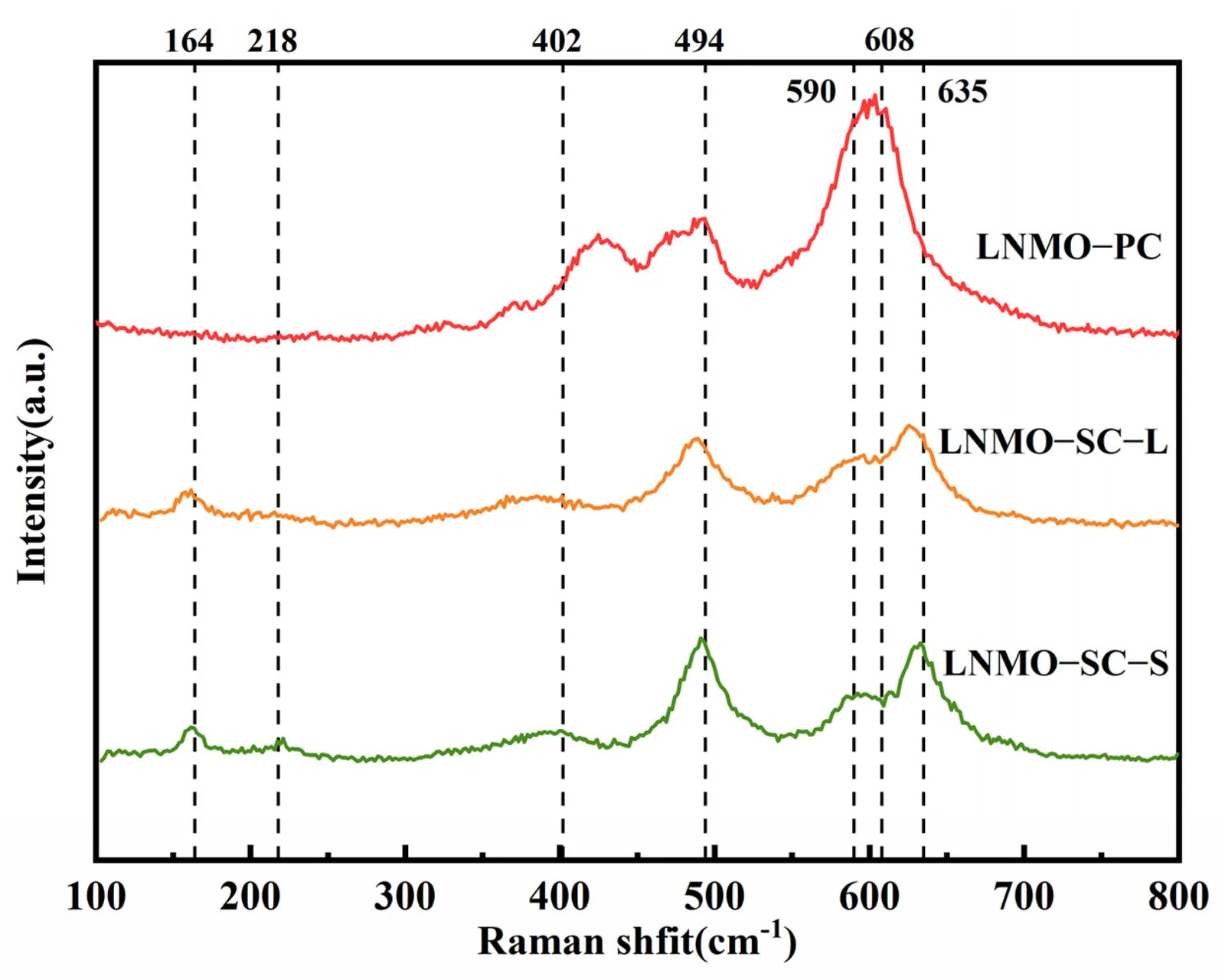
Raman spectra of LNMO-SC-S, LNMO-SC-L, and LNMO-PC.

Raman spectroscopy revealed the short-range atomic arrangement and biphasic structural characteristics of LNMO-PC (secondary polycrystalline aggregates), LNMO-SC-L (micron-sized primary particles), and LNMO-SC-S (submicron primary particles) (Figure 4). The peaks at 494 cm^−1^ and 635 cm^−1^ correspond to the stretching vibrations of Ni-O bonds and Mn-O bonds in the MnO_6_ octahedra, respectively, which are characteristic peaks of the Fd-3m disordered spinel phase, indicating the presence of the Fd-3m space group in all three samples [27]. The peaks at 164 cm^−1^, 218 cm^−1^, 402 cm^−1^, 590 cm^−1^, and 608 cm^−1^ correspond to the characteristic vibrational modes of the P4_3_32 ordered spinel phase [28]. From the spectrum, LNMO-PC shows almost no characteristic peaks of the P4_3_32 phase, with the Fd-3m disordered phase being dominant. The intensity of the A_1_g peak at 608 cm^−1^ is very high and accompanied by an obvious shoulder and peak broadening, reflecting the presence of a large number of oxygen vacancies and rock salt phase impurities in the lattice. The symmetry of the MnO_6_ octahedra is disrupted, leading to the appearance of additional vibrational modes that overlap with the A_1_g peak, causing an increase in peak intensity, peak broadening, and the appearance of a shoulder. In primary particle LNMO, which is highly ordered and has low defects, the vibrational modes of the MnO_6_ octahedra are highly singular, with no additional signals overlapping. As a result, the A_1_g peak intensity is lower, but the peak shape is sharp, and it may even be visually undetectable due to its weak signal. Both LNMO-SC-L and LNMO-SC-S clearly show the characteristic peaks of the P4_3_32 phase at 164 cm^−1^, 218 cm^−1^, 402 cm^−1^, and 590 cm^−1^, indicating the presence of significant ordered spinel phases in the primary particles. The P4_3_32 phase peaks in LNMO-SC-S are sharper and more stable, further confirming that its structure is the most ordered and has the highest crystallinity. The P4_3_32 phase peaks in LNMO-SC-L are slightly weaker, reflecting a lower degree of order compared to LNMO-SC-S. This result is consistent with the XRD characterization conclusion, clearly demonstrating the structural differences between secondary polycrystalline aggregates and primary particles in terms of bonding dimensions.

**Figure 5:**
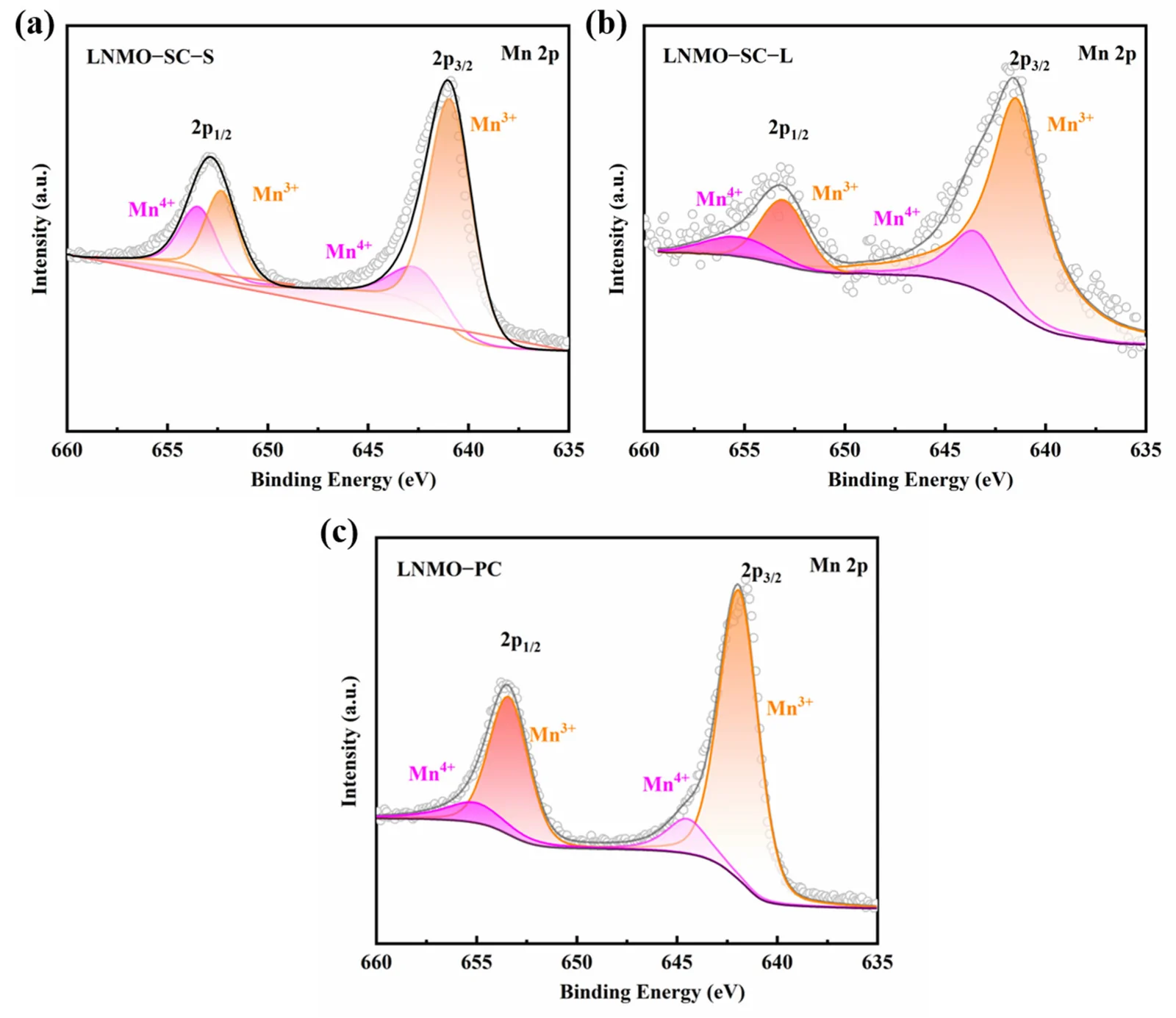
XPS spectra of (**a**) LNMO-SC-S [23] (reprinted from Journal of Alloys and Compounds, Vol. 971, Liu J, et al., Influence of different raw materials on the preparation of single-crystal LiNi_0.5_Mn_1.5_O_4_, Page 172778, Copyright (2024), with permission from Elsevier), (**b**) LNMO-SC-L, and (**c**) LNMO-PC.

Figure 5 shows the Mn 2p XPS fine spectra of the three LNMO samples. After Gaussian–Lorentzian curve fitting of the Mn 2p_3_/_2_ and 2p_1_/_2_ peaks, the relative atomic percentages of Mn^4+^ and Mn^3+^ are quantitatively determined: LNMO-SC-S contains 28.07% Mn^4+^ and 71.93% Mn^3+^, LNMO-SC-L contains 24.33% Mn^4+^ and 75.67% Mn^3+^, and LNMO-PC contains 19.68% Mn^4+^ and 80.32% Mn^3+^. The proportion of Mn^4+^ decreases in the order LNMO-SC-S > LNMO-SC-L > LNMO-PC, whereas Mn^3+^ follows the reverse sequence. It is widely recognized that excessive Mn^3+^ in LNMO triggers disproportionation reactions, producing soluble Mn^2+^ that dissolves into the electrolyte and leads to irreversible capacity loss. Secondary polycrystalline aggregate LNMO-PC is filled with dense grain boundaries, which induce severe Li/Ni cation mixing and abundant oxygen vacancies. These lattice defects drive the transformation of large amounts of Mn^4+^ into Mn^3+^, leading to the highest Mn^3+^ fraction and the most serious Mn dissolution issue. In contrast, primary particles eliminate such defect-induced valence deviation of manganese. The highest Mn^4+^ content of LNMO-SC-S stabilizes the spinel lattice and facilitates the formation of a protective CEI film on the particle surface to restrain Mn leaching. Meanwhile, its moderate Mn^3+^ derived from mild octahedral Ni/Mn disorder enlarges the lattice channels for Li^+^ migration, improving rate performance without aggravating Mn disproportionation. LNMO-SC-L exhibits intermediate Mn^4+^ and Mn^3+^ contents, so its structural stability and electrochemical performance lie between the other two samples, consistent with all foregoing characterization results.

**Figure 6:**
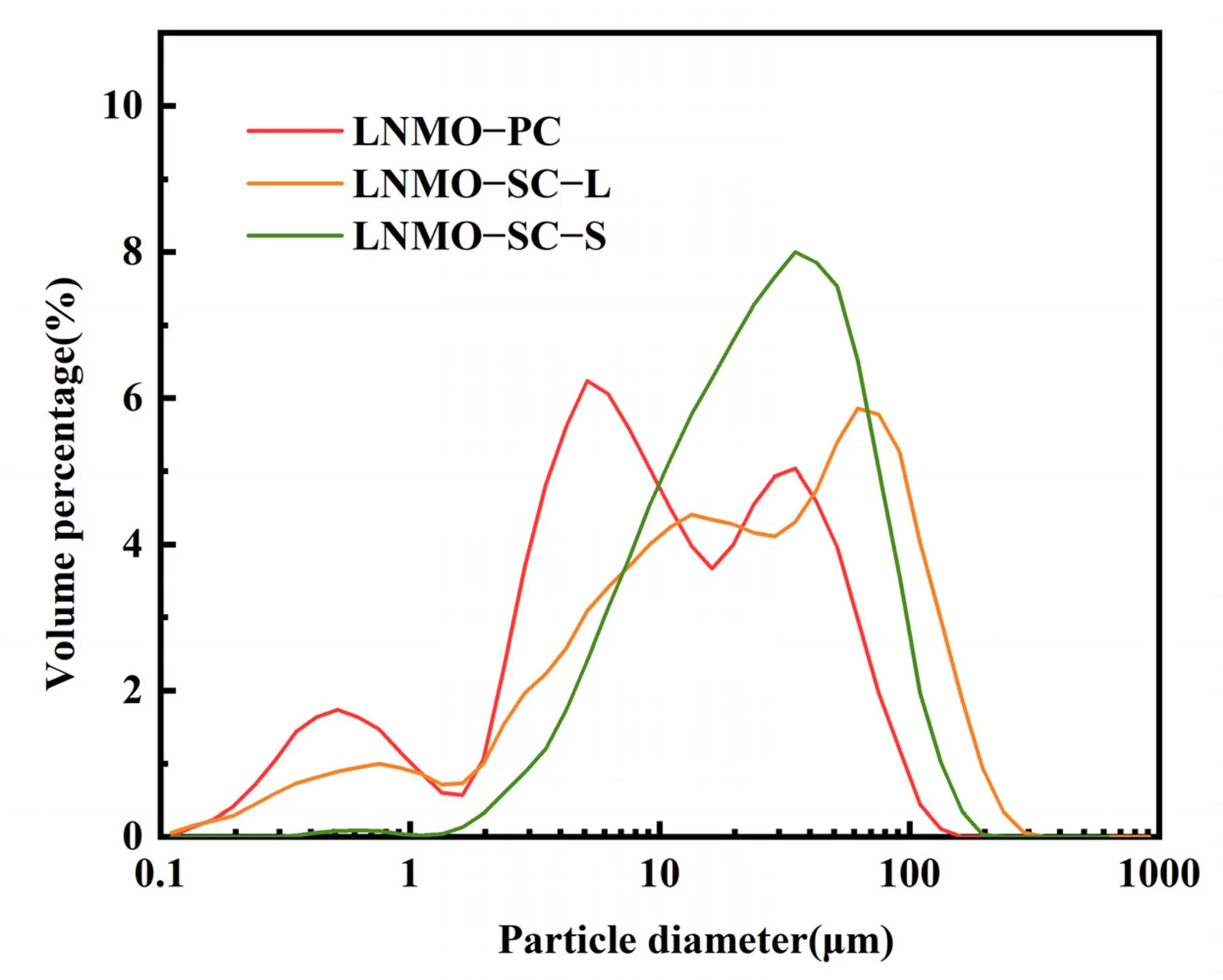
Particle size distribution curves of LNMO-SC-S, LNMO-SC-L, and LNMO-PC.

The particle size distribution curves of the three LNMO samples show significant differences (Figure 6). Horizontal coordinates denote particle diameter, and vertical coordinates represent volume fraction; peak height reflects the proportion of particles at corresponding sizes. Laser scattering measures hydrodynamic agglomerate size in liquid, differing from primary particle morphology observed via SEM. The measured volume median diameters (D50) are 8.547 μm for LNMO-PC, 21.35 μm for LNMO-SC-L, and 25.77 μm for LNMO-SC-S, matching the order of dominant distribution peaks. Only LNMO-SC-S presents a unimodal curve, while LNMO-PC and LNMO-SC-L show multi-modal distributions induced by particle fragmentation and uneven agglomeration. As defined in the Introduction, primary particles LNMO-SC-S and LNMO-SC-L only form weak agglomerates during testing, whereas loosely stacked secondary polycrystalline aggregate LNMO-PC easily breaks into fine submicron grains under ultrasonication. The LNMO-PC sample exhibits a multi-modal distribution with wide size span and low uniformity. Its multiple peaks stem from fragmented microcrystals and incomplete spherical aggregates, dispersing particle volume across a broad range and lowering the main peak height. The broad distribution brings excessive specific surface area, aggravating electrolyte side reactions and interfacial resistance, thus deteriorating cycling and rate performance. The LNMO-SC-L sample has a narrower distribution with faint secondary peaks derived from slight agglomerate separation. Large octahedral crystals guarantee structural integrity yet lengthen Li^+^ diffusion paths to slow ion transport. Uneven agglomeration leads to its lower D50 (21.35 μm) and weaker peak intensity relative to LNMO-SC-S. The LNMO-SC-S sample possesses the narrowest, most uniform size distribution with D50 of 25.77 μm and the highest dominant peak. Intact crack-free octahedral crystals resist ultrasonic fragmentation, so agglomerates concentrate within a narrow size range to form a sharp unimodal peak. SEM shows that its discrete primary octahedra are smaller than LNMO-PC spherical aggregates; the lack of fine broken grains increases its hydrodynamic median diameter. This balanced particle size shortens Li^+^ diffusion pathways and suppresses side reactions, enabling optimal cycling stability and rate capability.

Based on the ICP-OES mass concentration results above, the molar ratios of Li, Ni, and Mn were further calculated. The actual Li/Ni/Mn molar ratios are listed as follows: LNMO-SC-S (1:0.502:1.503), LNMO-SC-L (1:0.508:1.526), and LNMO-PC (1:0.505:1.514). All three samples exhibit negligible deviations from the ideal stoichiometric ratio 1:0.5:1.5 of LiNi_0.5_Mn_1.5_O_4_. The complete ICP mass concentration data and calculated molar stoichiometry are summarized in Table 3. It can be clearly seen that the mass contents of Ni and Mn among the three samples show only tiny fluctuations, and their actual molar ratios are all close to the theoretical stoichiometric ratio Ni/Mn = 1:3. Meanwhile, the lithium content of each sample only presents slight deviation without obvious divergence. Overall, the elemental stoichiometric deviation of Li, Ni, and Mn in all three materials is negligible. This result eliminates inconsistent elemental proportion as a variable responsible for the distinctions in crystal structure and electrochemical performance of the samples.

### 3.2. Electrochemical Performance

**Figure 7:**
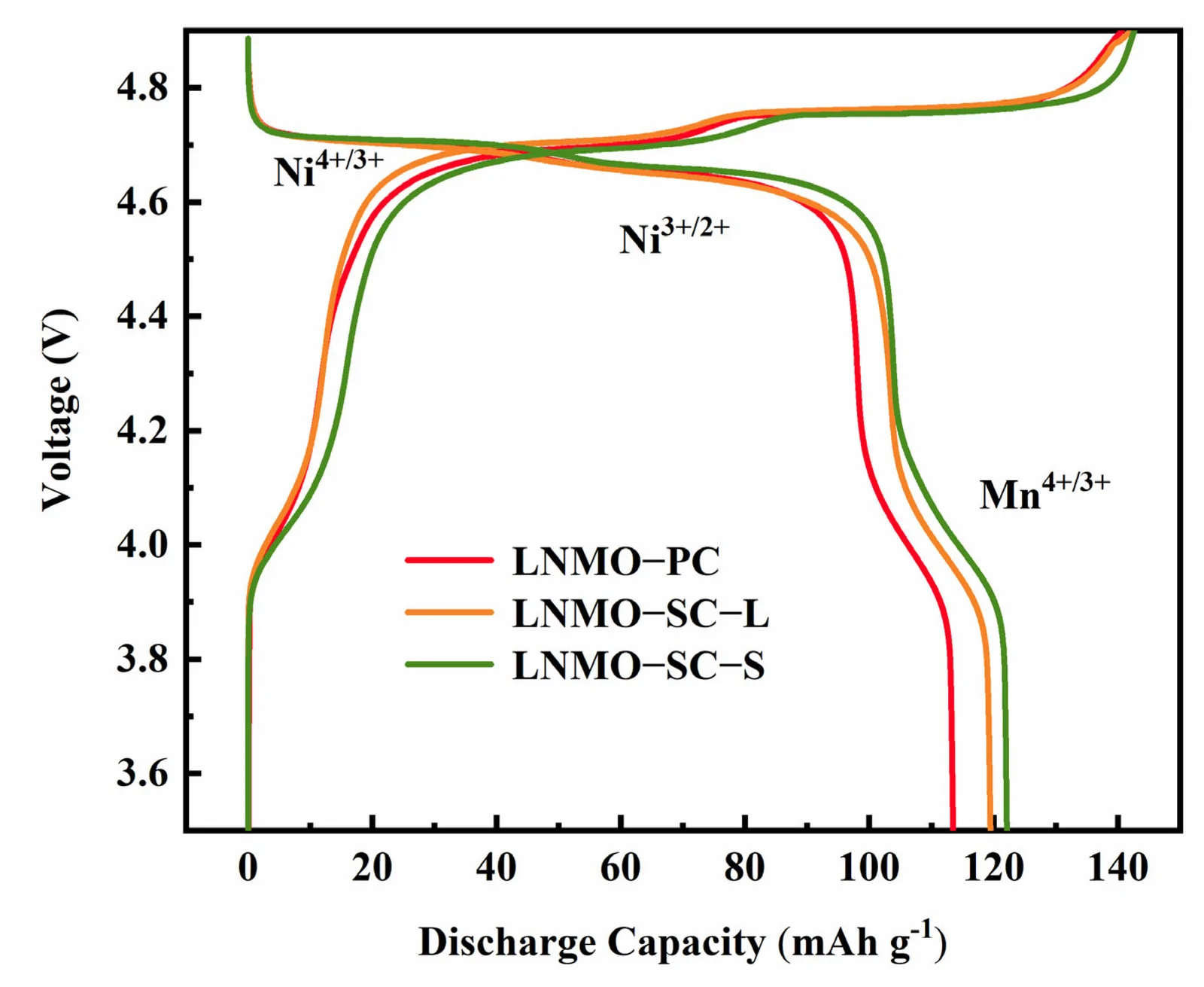
First charge–discharge curves of LNMO-SC-S, LNMO-SC-L, and LNMO-PC.

Figure 7 shows the first-cycle discharge curves of the three LNMO samples. The inflection points at approximately 4.7 V and 4.0 V correspond to the Ni^3+^/Ni^4+^, Ni^2+^/Ni^3+^, and Mn^3+^/Mn^4+^ redox couples, respectively [29], indicating that all three samples have the spinel-type LNMO structure with the Fd-3m space group. Regarding discharge capacity, LNMO-SC-S has the highest discharge capacity (approximately 122.14 mAh g^−1^) and the most stable voltage plateau. LNMO-SC-L has the second-highest capacity (approximately 119.50 mAh g^−1^), while LNMO-PC has the lowest capacity (approximately 113.48 mAh g^−1^), and the voltage drops more rapidly in the high-capacity region, indicating more irreversible capacity loss during the first cycle. When analyzing the first-cycle charge/discharge efficiency, the submicron primary particle (single-crystal morphology) LNMO-SC-S has the highest efficiency at 85.62%, suggesting it has the most reversible Li^+^, which is closely related to its small particle size, high crystallinity, and good dispersion achieved through the sol–gel method. These features effectively shorten the Li^+^ diffusion path and enhance electron transport efficiency. The submicron primary particle LNMO-SC-L limits Li^+^ diffusion, resulting in slightly lower performance, with a first-cycle efficiency of 84.12%. The secondary polycrystalline aggregate LNMO-PC has the lowest efficiency at 80.69% due to the higher grain boundary resistance, which reduces reversibility and capacity. In conclusion, reducing particle size and optimizing crystallinity are effective ways to improve the first-cycle charge/discharge efficiency of LNMO [30].

The long pseudo-plateau centered at approximately 4.7 V mainly corresponds to the two-electron redox process of Ni^2+^/Ni^4+^. As pointed out by previous crystallographic investigations on spinel LiNi_0.5_Mn_1.5_O_4_, this extended flat potential region is intrinsically associated with the coexistence of two crystalline phases with different lattice parameters during Li^+^ deintercalation rather than only originating from the single Ni redox pair. During high-voltage lithium extraction, continuous oxidation of Ni^2+^ to Ni^4+^ triggers lattice shrinkage, forming Li-rich and Li-poor spinel domains with distinct unit cell dimensions that coexist over a wide potential window; this biphasic equilibrium maintains a stable voltage and generates the prominent 4.7 V pseudo-plateau, accompanied by obvious spinel lattice rearrangement. The conversion from Ni^2+^ to Ni^4+^ breaks the electrostatic equilibrium of the MnO_6_ octahedral framework, causing subtle unit cell fluctuation and local rearrangement of Li and transition metal ions at tetrahedral and octahedral sites; such synchronous structural distortion, cation rearrangement and biphasic coexistence jointly generate the prominent pseudo-plateau near 4.7 V. The minor short plateau at ~4.0 V belongs to the weak Mn^3+^/Mn^4+^ redox pair and exerts little influence on the main lattice evolution and biphasic transition behavior.

Combined with the Rietveld refinement results, the structural reversibility of this high-voltage biphasic transition differs distinctly among three samples. LNMO-SC-S shows the highest I_311_/I_400_ value and negligible rock-salt impurities, indicating highly ordered occupation of Li tetrahedral sites and primary particles with sparse internal grain boundaries. This well-ordered, low-defect lattice effectively suppresses irreversible lattice mismatch and strain during the Li-rich/Li-poor biphasic transformation upon Ni redox, endowing the 4.7 V pseudo-plateau with excellent structural reversibility and stable biphasic cycling behavior. By contrast, LNMO-PC possesses abundant internal grain boundaries and severe Li/Ni tetrahedral mixing, as reflected by its lowest I_311_/I_400_ ratio and the largest amount of rock-salt secondary phase. These dense structural defects amplify lattice parameter mismatch between the two coexisting phases during repeated delithiation/lithiation cycles, readily accumulating irreversible lattice strain during the high-voltage biphasic structural transformation, which severely impairs the reversibility of the 4.7 V redox pseudo-plateau and accelerates capacity decay.

**Figure 8:**
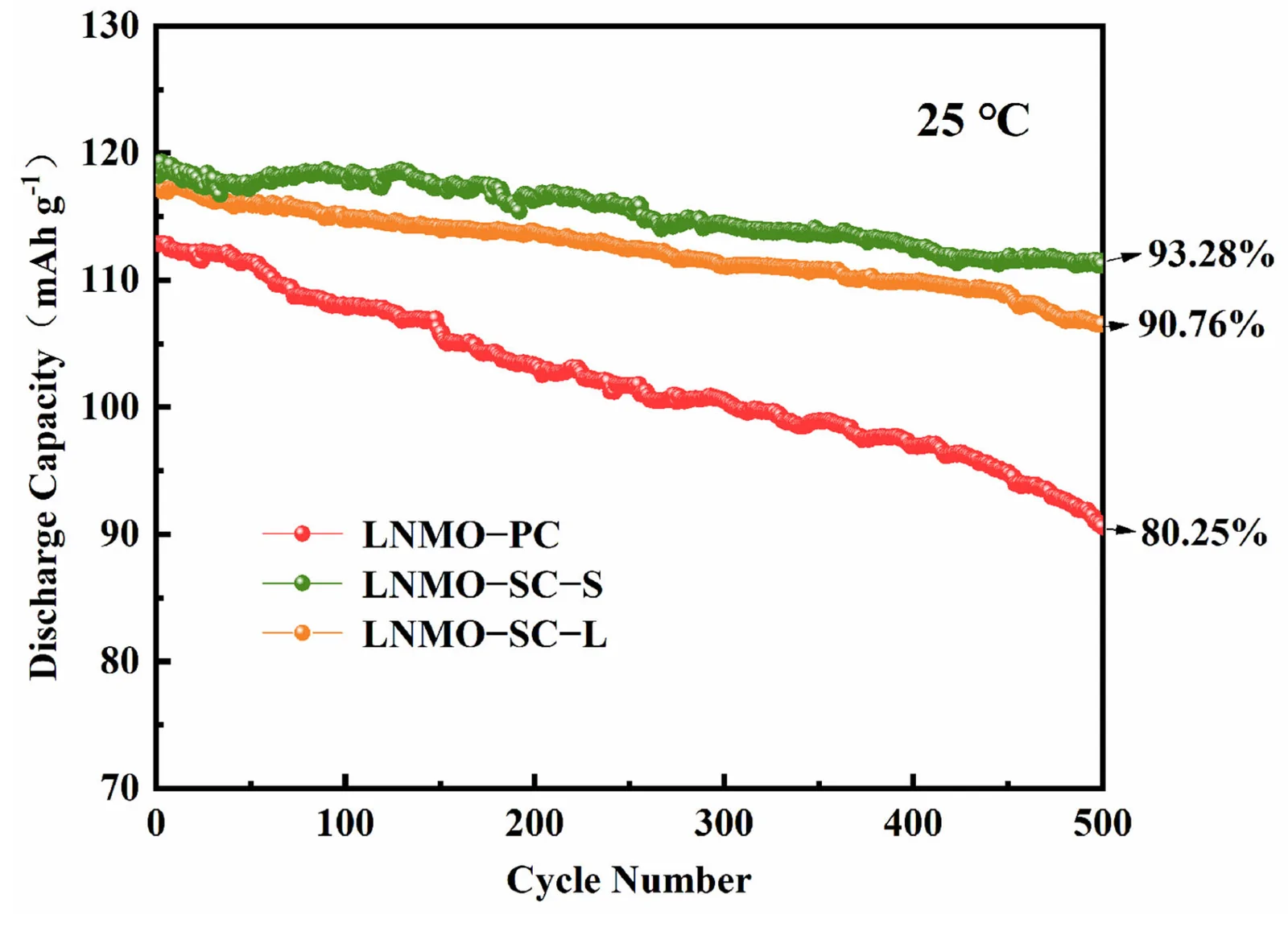
Cycling performance of LNMO-SC-S, LNMO-SC-L, and LNMO-PC at 1 C.

Figure 8 shows the long-cycle performance curves of secondary polycrystalline aggregate LNMO-PC prepared by the co-precipitation method, micron-sized primary particle LNMO-SC-L prepared by the molten salt method, and submicron primary particle LNMO-SC-S prepared by the sol–gel method at a 1 C rate and 25 °C. After 500 cycles, the capacity retention rates of the three samples were 80.25%, 90.76%, and 93.28%, corresponding to discharge capacities of approximately 90.58 mAh g^−1^, 106.51 mAh g^−1^, and 111.19 mAh g^−1^, respectively. Among them, the submicron primary particle LNMO-SC-S exhibited the highest capacity retention and the most gradual capacity decay during cycling, which is closely related to the small particle size, high crystallinity, and good dispersion achieved through the sol–gel method. These factors effectively shorten the Li^+^ diffusion path [1], alleviate structural stress during cycling, and suppress grain boundary cracking and phase separation, significantly enhancing cycling stability. Although the micron-sized primary particle LNMO-SC-L exhibited relatively good structural stability, its larger particle size somewhat limited Li^+^ diffusion efficiency, and internal stress was more likely to accumulate during long cycles, leading to a slightly lower capacity retention compared to the submicron primary particles. The secondary polycrystalline aggregate LNMO-PC, due to the presence of many grain boundaries, is prone to micro-crack propagation and structural fragmentation at the grain boundaries during repeated lithium intercalation/deintercalation, leading to loss of contact between the active material and the conductive network, resulting in rapid capacity decay.

**Figure 9:**
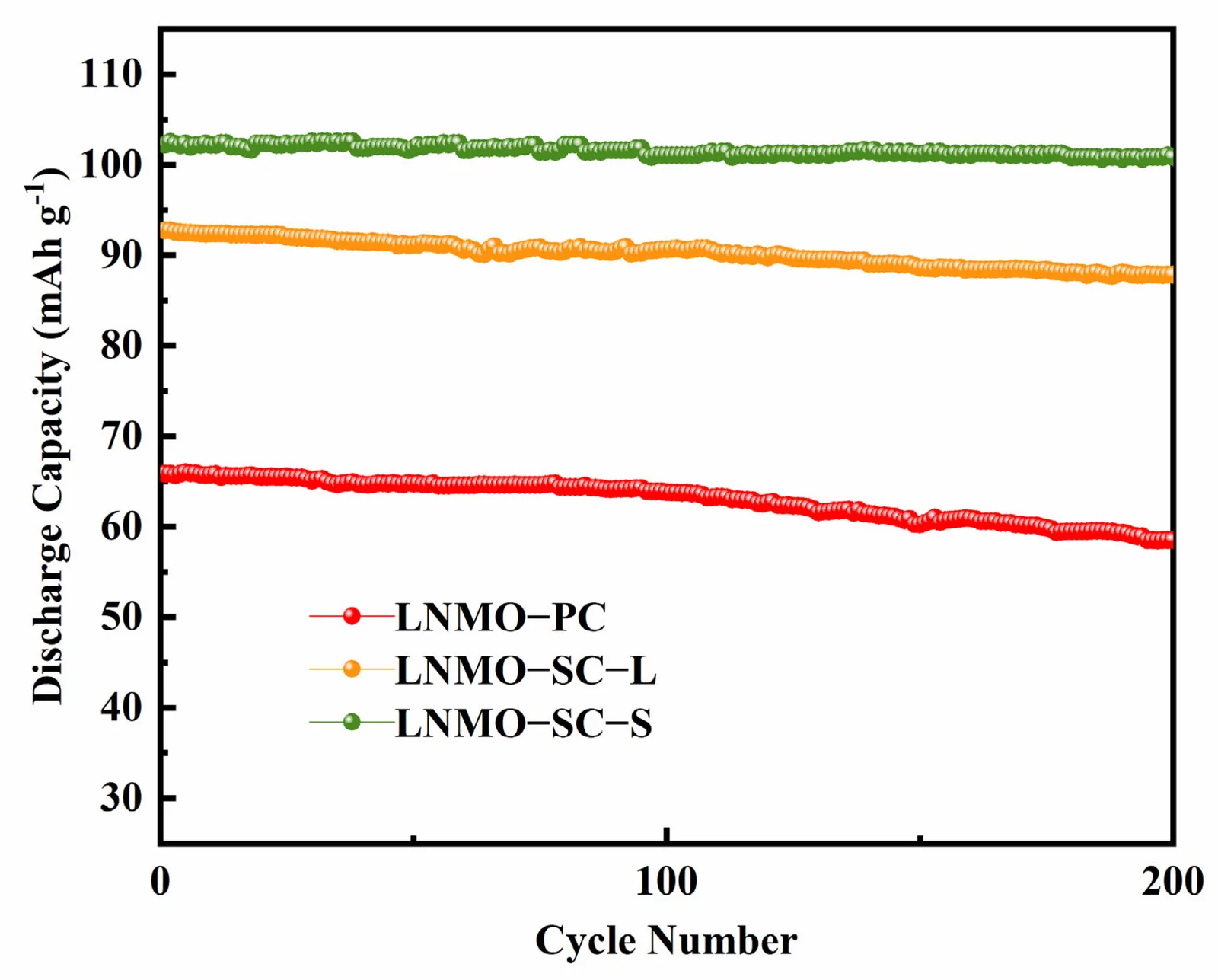
Cycling performance of LNMO-SC-S, LNMO-SC-L, and LNMO-PC at 5 C.

Figure 9 shows the cycling performance curves of three LNMO samples—LNMO-PC, LNMO-SC-L, and LNMO-SC-S—at a 5 C rate and 25 °C. As shown in the figure, there are significant differences in the discharge capacity and cycling stability of the three samples at high rates: LNMO-SC-S exhibits the best high-rate performance, with an initial discharge capacity of approximately 102 mAh g^−1^, and after 200 cycles, its capacity remains stable with almost no noticeable decay. LNMO-SC-L has an initial discharge capacity of about 92 mAh g^−1^, with a slight decrease in capacity during cycling, but the capacity generally stays around 90 mAh g^−1^. In contrast, LNMO-PC has an initial discharge capacity of only about 65 mAh g^−1^, and its capacity continuously declines as cycling progresses, dropping to about 58 mAh g^−1^ after 200 cycles, exhibiting the worst high-rate performance. The submicron primary particle (single-crystal morphology) LNMO-SC-S benefits from the small particle size, high crystallinity, and good dispersion achieved by the sol–gel method, which effectively shortens the Li^+^ diffusion path and enhances electron transport efficiency. As a result, it can still complete the lithium intercalation/deintercalation process quickly at a 5 C high rate, while alleviating structural stress and suppressing capacity decay, showing the best high-rate cycling performance. The micron-sized primary particle (single-crystal morphology) LNMO-SC-L maintains structural integrity, but its larger particle size somewhat limits the Li^+^ diffusion rate, leading to slightly lower initial capacity at high rates compared to the submicron primary particles. However, its structure still effectively suppresses grain boundary cracking, maintaining good cycling stability. The secondary polycrystalline aggregate LNMO-PC, due to the presence of many grain boundaries, faces significantly increased Li^+^ diffusion resistance at high rates, leading to a lower initial capacity. Additionally, during repeated lithium intercalation/deintercalation, micro-cracks tend to form at the grain boundaries, accelerating structural fragmentation and capacity decay, resulting in the worst high-rate cycling performance.

**Figure 10:**
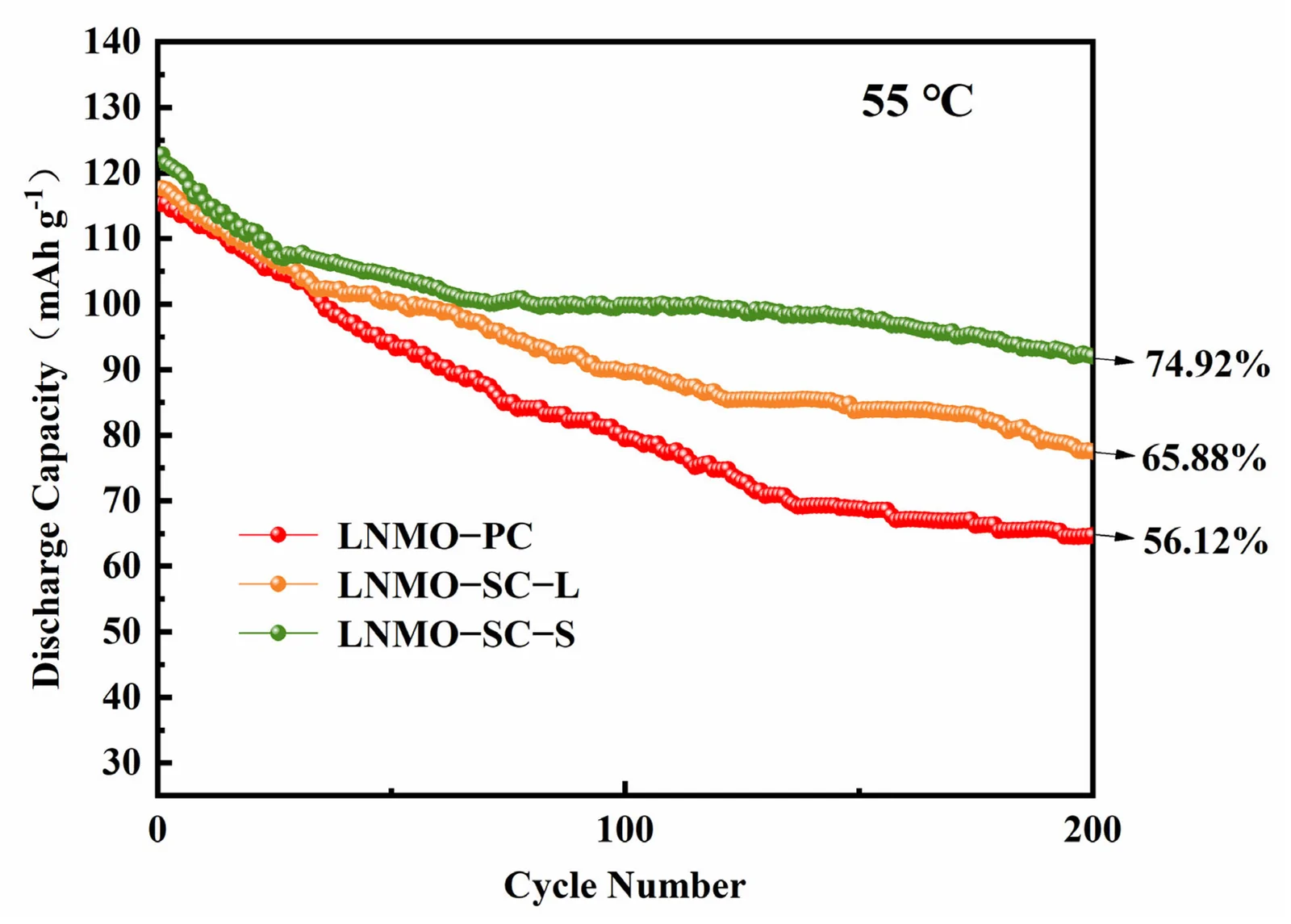
LNMO-SC-S, LNMO-SC-L, and LNMO-PC cycling performance at 55 °C and 1 C.

Figure 10 shows the cycling performance curves of three LNMO samples—LNMO-PC, LNMO-SC-L, and LNMO-SC-S—at a 1 C rate and 55 °C. After 200 cycles, the capacity retention rates of the three samples were 56.12%, 65.88%, and 74.92%, respectively, showing significant differences in high-temperature stability. Among them, the submicron primary particle LNMO-SC-S had the highest capacity retention and the best high-temperature cycling stability. The small particle size and high crystallinity of LNMO, prepared by the sol–gel method, significantly alleviate structural stress at high temperatures and suppress Mn^3+^ disproportionation and Mn^2+^ dissolution, thereby delaying material degradation and capacity fading. The micron-sized primary particle LNMO-SC-L is more likely to accumulate internal stress at high temperatures, resulting in a slightly lower capacity retention than the submicron primary particles. On the other hand, the secondary polycrystalline aggregate LNMO-PC, due to the presence of many grain boundaries, is more susceptible to micro-crack propagation and structural fragmentation at the grain boundaries under the combined effects of high temperature and repeated lithium intercalation/deintercalation. This accelerates Mn dissolution and electrolyte decomposition, leading to the deactivation of active materials, rapid capacity fading, and the poorest high-temperature stability.

**Figure 11:**
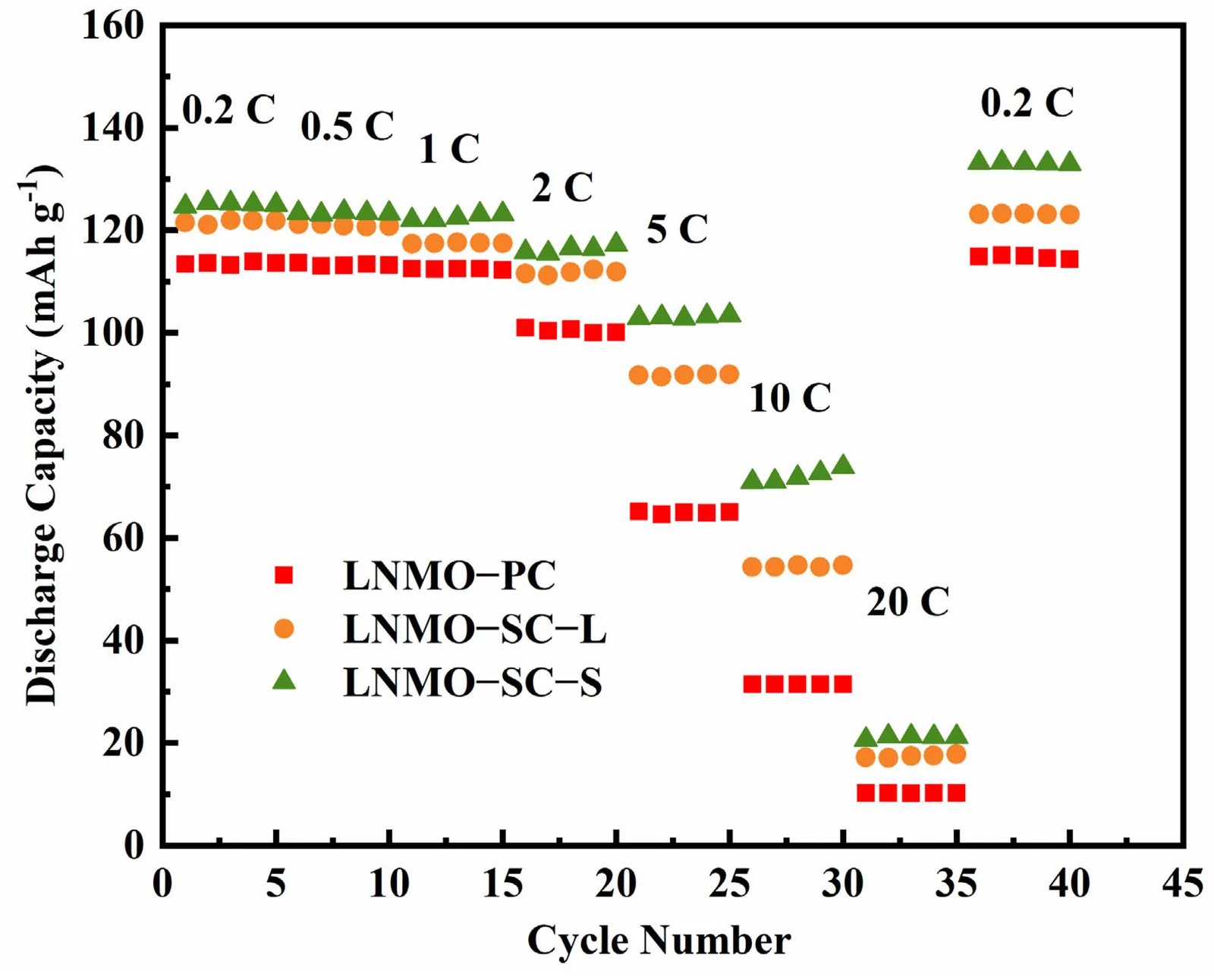
Rate performance of LNMO-SC-S, LNMO-SC-L, and LNMO-PC.

As seen in Figure 11, there are significant differences in the discharge capacity and rate tolerance of the three samples at different rates. LNMO-SC-S exhibits the highest discharge capacity at all test rates and the most gradual capacity decay. At 0.2 C, the capacity is approximately 130 mAh g^−1^, and at 20 C, it still maintains about 20 mAh g^−1^, demonstrating the best high-rate performance. LNMO-SC-L shows intermediate rate performance, with capacity slightly lower than LNMO-SC-S at all rates; at 20 C, the capacity is about 15 mAh g^−1^. LNMO-PC exhibits the poorest rate performance, with the capacity at all rates being the lowest among the three samples, and capacity decay is particularly severe at high rates (≥10 C), with the capacity dropping to about 10 mAh g^−1^ at 20 C. This performance difference can be attributed to the structural characteristics brought about by different morphologies and preparation methods: the submicron primary particle LNMO-SC-S, with its small particle size achieved through the sol–gel method, enhances electron transport efficiency, enabling it to rapidly complete the lithium intercalation/deintercalation process at high rates and thus exhibiting the best rate performance. The micron-sized primary particle LNMO-SC-L somewhat limits the Li^+^ diffusion rate, resulting in slightly lower capacity at high rates compared to the submicron primary particles. The secondary polycrystalline aggregate LNMO-PC, due to the presence of many grain boundaries, faces significantly increased Li^+^ diffusion resistance at high rates, leading to rapid capacity decay and thus the worst rate performance.

**Figure 12:**
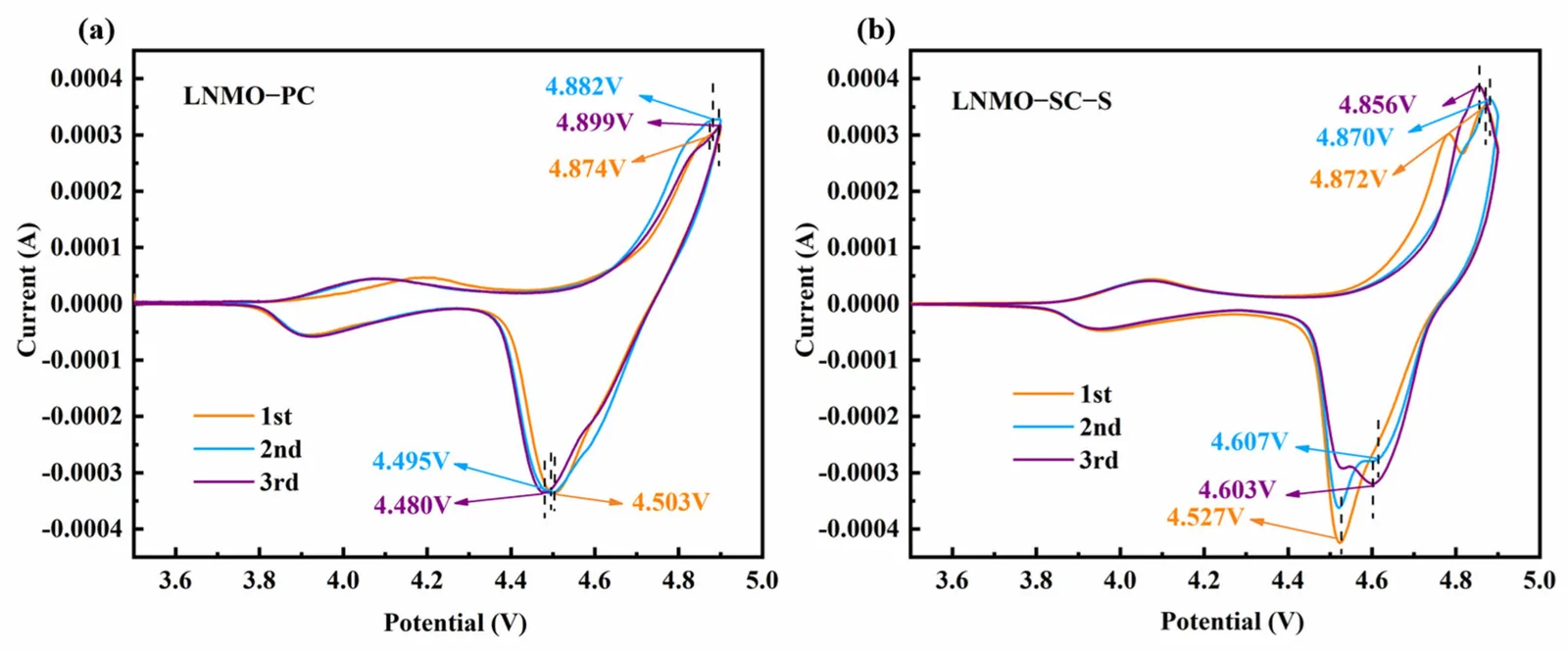
CV curves for (**a**) LNMO-PC and (**b**) LNMO-SC-S during the first three cycles [22]. (Reprinted from Ceramics International, Vol. 48, Liu J, et al., Improving the electrochemical performance of single crystal LiNi_0.5_Mn_1.5_O_4_ cathode materials by Y–Ti doping and unannealing process, Pages 36490–36499, Copyright (2022), with permission from Elsevier).

Figure 12a,b show the first three cycles of cyclic voltammetry (CV) curves for secondary polycrystalline aggregate LNMO-PC prepared by the co-precipitation method and submicron primary particle LNMO-SC-S prepared by the sol–gel method, respectively. The two main peaks around 4.7 V correspond to the Ni^2+^/Ni^3+^ and Ni^3+^/Ni^4+^ redox couples, indicating that both samples have the spinel-type LNMO structure with the Fd-3m space group. The small peak around 4.0 V is associated with the Mn^3+^/Mn^4+^ redox couple [29]. The potential difference (ΔE = Epa − Epc) between the oxidation and reduction peaks can be used to characterize the degree of electrochemical polarization. The smaller the ΔE value, the better the reversibility of Li^+^ insertion/extraction and the lower the polarization. According to the Figure 12 and Table 4, the ΔE value for LNMO-SC-S is significantly lower than that of LNMO-PC during the first three cycles, and it continues to decrease and stabilize as the number of cycles increases. In contrast, the ΔE value for LNMO-PC fluctuates more and is generally higher. This suggests that LNMO-SC-S has better Li^+^ insertion/extraction reversibility and lower electrochemical polarization, further proving that submicron primary particles can effectively reduce polarization during battery cycling. This may be due to the small particle size, high crystallinity, and good dispersion of the submicron primary particles, which enhances the material’s conductivity, shortens the Li^+^ diffusion path [31], and thus improves reaction kinetics and structural stability.

**Figure 13:**
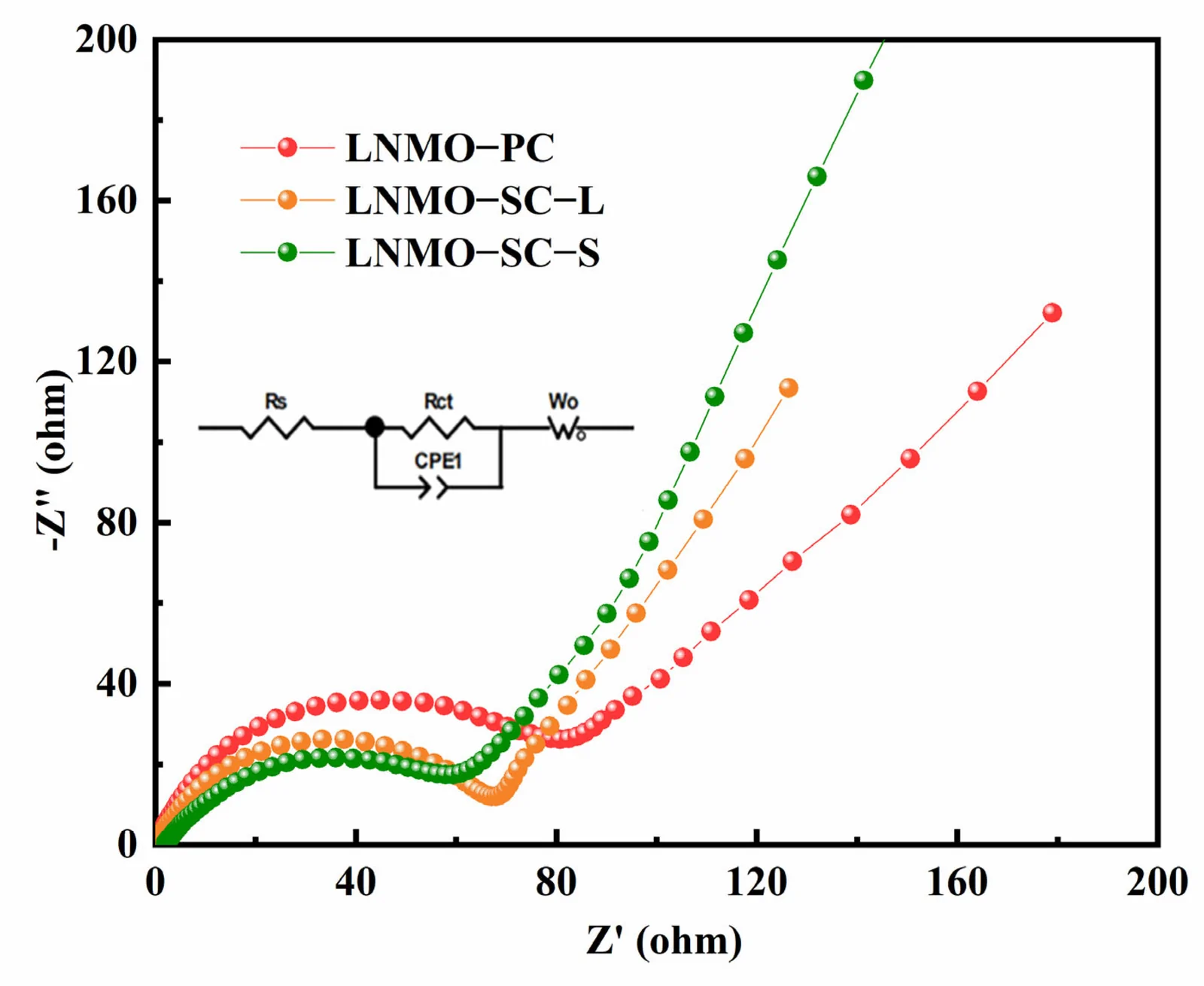
LNMO-SC-S, LNMO-SC-L, and LNMO-PC Nyquist curves after 200 cycles.

Figure 13 shows the electrochemical impedance spectra of the three LNMO samples. The Nyquist plot consists of a high-frequency semicircle and a low-frequency sloping line. After fitting with ZView, the Rct is the charge transfer resistance. The fitting results show that LNMO-SC-S has the smallest Rct (57.29 Ω), the shortest semicircle diameter, and the highest low-frequency slope, indicating the lowest charge transfer resistance and the fastest Li^+^ diffusion kinetics. LNMO-SC-L has a moderate Rct (59.83 Ω), while LNMO-PC has the largest Rct (89.57 Ω), showing significantly higher charge transfer resistance. This difference is attributed to the submicron primary particles effectively shortening the Li^+^ diffusion path and enhancing electron transport efficiency, while the numerous grain boundaries in the secondary polycrystalline aggregates increase the charge transfer resistance.

The submicron primary particle LNMO-SC-S possesses continuous, integrated crystal domains with negligible internal grain boundaries. The absence of massive grain boundaries effectively relieves cyclic lattice strain and restrains the generation of Jahn–Teller-active Mn^3+^, which fundamentally suppresses Mn disproportionation and irreversible manganese dissolution during repeated lithium insertion/extraction. Meanwhile, its uniform small particle size shortens solid-state Li^+^ migration distance, accelerates interfacial charge transfer kinetics, and reduces overall electrochemical polarization, as confirmed by the lowest fitted Rct value after long-term cycling.

In contrast, spherical secondary polycrystalline aggregate LNMO-PC is assembled from countless primary crystallites, creating abundant internal grain boundaries. These grain boundaries serve as defect sites that aggravate cation disorder, raise Mn^3+^ proportion, and introduce severe lattice distortion. During cycling, crack propagation along grain boundaries destroys structural integrity, triggers persistent interfacial side reactions, and causes rapid capacity decay. The micron-sized primary particle LNMO-SC-L avoids dense grain boundaries yet suffers from elongated lithium diffusion paths due to enlarged particle dimension, limiting its high-rate reaction kinetics.

## 4. Conclusions

This study addresses the key issues of structural instability, rapid capacity decay, and poor high-temperature and rate performance of high-voltage spinel LiNi_0.5_Mn_1.5_O_4_ (LNMO) cathode materials. Secondary polycrystalline aggregate LNMO-PC, micron-sized primary particle LNMO-SC-L, and submicron primary particle LNMO-SC-S samples were prepared using co-precipitation, molten salt, and sol–gel methods, respectively. The study systematically investigates the mechanism by which particle morphology influences the crystal structure, interface chemical states, and electrochemical performance of the materials. Structural characterization results show that submicron primary particle (single-crystal morphology) LNMO-SC-S exhibits the highest crystallinity, the least impurity phases and lattice defects, and the highest Mn^4+^ content, which effectively suppresses the Mn^3+^ disproportionation reaction. In contrast, secondary polycrystalline aggregates exhibit significant structural instability due to dense grain boundaries, and micron-sized primary particles (single-crystal morphology) are limited by particle size, with room for improvement in kinetic performance. Electrochemical tests reveal that LNMO-SC-S, benefiting from the synergistic advantages of the primary particle, low grain-boundary structure, and small particle size, exhibits the best performance in long-cycle stability at room temperature, high-temperature stability, and high-rate performance. It also has the lowest charge transfer resistance and electrochemical polarization. This study clarifies the intrinsic structure–performance relationship of LNMO materials and confirms that primary particle and small particle size are effective approaches to improving the overall electrochemical performance of LNMO.

## Figures and Tables

**Table 1:** Comparative table of charge–discharge performance between LNMO-SC-S and similar materials reported in references.

	Cycle Stability	R_ct_ Value	Cycle Stability at 55 °C	Reference
LNMO-SC-S (submicron primary particles (single-crystal morphology)), this work)	1 C, 500 cycles: 111.19 mAh g^−1^, retention 93.28% 5 C, 200 cycles: ~102 mAh g^−1^, negligible decay	57.29 Ω (after 200 cycles)	1 C, 200 cycles, 92 mAh g^−1^, retention 74.92%	This article
LNMO@ Ta_2_O_5_	0.2 C, 100 cycles: 125.62 mAh g^−1^, retention 97%		0.1 C, 100 cycles: 122.3 mAh g^−1^, retention 93%	[18]
LNMO@ LiCoPO_4_	0.5 C, 100 cycles: 132 mAh g^−1^, retention 98.5%	122.1 Ω (before cycling)	0.5 C, 60 cycles: 130 mAh g^−1^	[19]
LNMO@ graphene	0.1 C, 100 cycles: 114.4 mAh g^−1^ 5 C, 500 cycles: 104.5 mAh g^–1^	54.6 Ω (after 100 cycles at 2 C)	100 cycles: 83.8 mAh g^−1^, retention 94.5%	[20]
LNMO@ LiPON solid electrolyte	0.4 C, 100 cycles: 119.02 mAh g^−1^, retention 97%	63.5 Ω (after 100 cycles)		[21]

**Table 2:** Lattice parameters and I_311_/I_400_ values of the three samples.

Samples	Lattice Parameters		I_311_/I_400_	R_wp_/%
	a/Å	V/Å^3^		
LNMO-SC-S	8.1189	535.17	1.0087	8.1
LNMO-SC-L	8.1274	536.85	0.9802	8.2
LNMO-PC	8.1282	537.01	0.6811	8.5

**Table 3:** Element mass concentrations and calculated Li/Ni/Mn molar stoichiometric ratios of three LNMO samples.

Samples	Li (mg/kg)	Ni (mg/kg)	Mn (mg/kg)	Li:Ni:Mn Molar Ratio
LNMO-SC-S	37,922	161,117	451,038	1:0.502:1.503
LNMO-SC-L	37,435	160,942	452,079	1:0.508:1.526
LNMO-PC	37,633	160,595	450,824	1:0.505:1.514

**Table 4:** The ΔE values of the first three cycles for LNMO-PC and LNMO-SC-S.

	LNMO-PC			LNMO-SC-S		
	Epa	Epc	ΔE	Epa	Epc	ΔE
1st	4.874	4.503	0.371	4.872	4.527	0.345
2nd	4.882	4.495	0.387	4.870	4.607	0.263
3rd	4.899	4.480	0.419	4.856	4.603	0.253

## Data Availability

The original contributions presented in this study are included in the article. Further inquiries can be directed to the corresponding authors.
